# A dietary modelling approach to examine the impact on vitamin C intakes in the United Kingdom with varying consumption of fruit juice

**DOI:** 10.3389/fnut.2026.1794774

**Published:** 2026-07-31

**Authors:** Alyson J. Hill, Emily Royle, Graham Horgan, Maeve A. Kerr

**Affiliations:** 1 Nutrition Innovation Centre for Food and Health (NICHE), School of Biomedical Sciences, Ulster University, Coleraine, United Kingdom; 2 Rowett Institute, University of Aberdeen, Aberdeen, United Kingdom

**Keywords:** dietary intakes, dietary modelling, fruit juice, NDNS national diet and nutrition survey, vitamin C

## Abstract

Vitamin C (Ascorbic acid) has many important roles in maintaining health and preventing disease. As a water-soluble vitamin, it cannot be synthesized endogenously or stored, therefore must be obtained regularly in sufficient amounts from the diet. The present study examined vitamin C intakes and contributors in a United Kingdom population and used dietary modelling to examine the impact of adding or replacing fruit juice (FJ) on vitamin C intakes in participants within the United Kingdom National Diet and Nutrition survey (NDNS 2016–2019; *n* = 3,558). Results indicated that 40% of this population consumed FJ which contributed to 25% of vitamin C intakes across all ages. Vitamin C from foods and drinks typically exceeded Reference Nutrient Intakes (RNI) (30–40 mg/day), although <10% of the population fell below the Estimated Average Requirement (EAR) and <2% were below lower RNI (LRNI). Theoretical modelling showed that increasing numbers of FJ consumers would increase mean intakes of vitamin C. However, a greater effect on the proportion meeting Dietary Reference Values (DRVs) for vitamin C was observed by broadening FJ consumption rather than by increasing the amounts consumed by existing consumers. This also had a minimal impact on free sugar intakes. Therefore encouraging more people to include a one daily 150 mL serving of FJ in their diet (as opposed to suggesting increased intake within current consumers), would positively impact on vitamin C intakes within the United Kingdom population, whilst also helping to achieve the national “5-a-day” recommendation via a convenient, accessible dietary choice.

## Introduction

Vitamin C (Ascorbic acid) has many important roles in the maintenance of health and prevention of disease (1). It is an essential co-factor required for biosynthesis of collagen, an important component of connective tissue which plays a vital role in wound healing (2). Vitamin C is a powerful antioxidant whereby it can neutralize reactive oxygen species and prevent oxidative stress and cellular damage (2), both of which are implicated in the development of chronic diseases such as cardiovascular disease and cancer (3). Additionally, vitamin C has a role in immune function by supporting various cellular functions, and iron absorption by increasing the gastrointestinal tract’s acidity, which enhances the conversion of nonabsorbable ferric iron (Fe^3+^) to its absorbable ferrous (Fe^2+^) state (2).

As vitamin C cannot be synthesized endogenously or stored, it must be obtained regularly in sufficient amounts from the diet (2). Fruits and vegetables are rich sources, especially citrus fruits, berries, spinach, tomatoes, and green leafy vegetables. Fruit juices (FJs) also contain a good source of vitamin C and one portion (150 mLs) can be recommended as a convenient option to help individuals achieve the dietary guideline alongside fresh fruit and vegetables. Notably, there is wide variation in the vitamin C content of fruits, vegetables and juices since vitamin C is water-soluble and, thus, sensitive to heat and oxygen, resulting in losses due to storage and cooking.

The recommended daily intake of vitamin C varies depending on the country. In the United Kingdom, the Reference Nutrient Intake (RNI) has been set at 30 and 40 mg/day for children and adults, respectively, (4). Achievement of the RNI is facilitated through adherence to national food-based guidelines recommending at least “5-a-day” fruit and vegetables daily (minimum 400 g/day), where one portion is approximately 80 g for adults and 40–60 g for children and expected to provide approximately 200 mg of vitamin C daily. However, adherence to the 5-a-day guideline is poor and only a small number of people achieve this target. The most recent National Diet and Nutrition Survey (NDNS) (collected 2019–2023) shows that, on average, United Kingdom adults consume just 3.3 to 3.7 portions of fruit and vegetables per day (depending on age) with fewer than 1 in 5 adults (17%) meeting the current 5-a-day target. Additionally, United Kingdom children aged 11 to 18 years typically consume just 2.8 portions of fruit and vegetables per day, with fewer than 1 in 10 (9%) meeting the target (5). United Kingdom guidelines recommend consuming a variety, where one portion of fruit includes types that are fresh, frozen, canned, dried and that a moderate consumption of 100% fruit juice (FJ) or smoothie (maximum 150 mLs) per day counts as one of the portions and can contribute towards the 5-a-day recommendation.

There are several key differences between the nutritional composition of 100% FJ and the whole fruit it is extracted from. Fibre content is modestly higher in whole fruit as it has been removed during the manufacturing process to produce the juice. However glycaemic index (GI) is similar, with both being classed as low GI (<55; average GI value of whole fruit: 51 vs. FJ: 47), meaning they are digested and absorbed more slowly than high GI foods (6), therefore having less impact on blood sugar levels. Additionally, both whole fruit and 100% FJ contain significant amounts of bioactive components such as flavanones, polyphenols and carotenoids which have favourable effects on health and are reportedly better absorbed from FJ as a consequence of the lower fibre levels (7). Whole fruits when consumed as a preload to a meal, are shown to lead to higher satiation and feeling of fullness compared to fruit consumed in the form of purees and juices, resulting in a lower energy intake. This may be due to the higher dietary fibre content in whole fruit contributing to slower gastric emptying, or due to structure and chewing since adding fibre to FJ did not change the result (8).

FJ is legally defined as the “fermentable but unfermented product obtained from the edible part of fruit” and cannot contain added sugars, sweeteners, flavours, preservatives, colours or water. In contrast to FJ, nectars and fruit drinks are permitted to contain added sugars and/or sweeteners as well as other ingredients, such as botanicals. There is no accepted definition for smoothies in European or United Kingdom law. A recent review (9) found that FJ contributed 4–26% to vitamin C intakes in European populations of different age ranges based on national survey data, as well as making small contributions to intakes of folate and potassium (up to 7%). The review also provided information on daily intakes of FJ in the United Kingdom, reporting a range of 34 g–63 g per day across age groups, inclusive of non-consumers.

Given the role of FJ in contributing towards one portion of daily fruit and vegetable recommendations, this study aimed to (1) examine vitamin C intakes and contributors in a representative sample of the United Kingdom population using NDNS data from 2016–2019 and (2) examine the effects of adding and/or replacing FJ on vitamin C intakes with fruit, other foods or other beverages. It is worth noting that the NDNS did not report vitamin C intakes between 2014–2025.

## Methods

### Design and study population

The NDNS which commenced in 2008, is a cross-sectional, annual rolling survey designed to collect detailed, quantitative information on food consumption, nutrient intake and nutritional status in the general United Kingdom population aged 1.5 years and older. NDNS uses a stratified sampling design to generate a random sample of private United Kingdom households each year and a nationally representative core sample of around 1,000 participants (500 adults, 500 children) each year. The design provides for additional recruitment at devolved country level. As the newest NDNS data were not available at the time of this analysis, the present study utilised food and nutrient intake data from years 9 to 11 (2016–2019; *n* 3,558) which were obtained from the United Kingdom Data Archive (NatCen, University of Essex, Colchester, Essex, United Kingdom). Ethical approval for the secondary analyses was not required as this study presents results using anonymized human data.

Full sampling and methodological details for years 9–11 of the NDNS are available elsewhere (10). In summary, food intake data were collected via an estimated, un-weighed food diary over 4 consecutive days. Days of food recording were randomly assigned with the aim to evenly represent all days of the week in the NDNS dataset each year. Completed paper food diaries were manually coded using a bespoke diet coding and analysis programme (Diet In, Nutrients Out, DINO). Coded foods and dietary supplements were cross-referenced with food composition data in the NDNS nutrient databank which draws on information from the Composition of Foods Integrated Dataset (CoFID), the Food Standards Agency Food Recipes Database, and manufacturers’ data gathered through food labels and web information.

### Survey and food composition database

NDNS population groups included in the current study were children 1.5–3 yrs. (*n* 306), children 4–10 yrs. (*n* 725), adolescents 11–14 yrs. (*n* 357) and 15–18 yrs. (*n* 326), and adults 19–64 yrs. (*n* 1,392), 65–74 yrs. (*n* 262) and 75 + yrs. (*n* 190). The age ranges were chosen to align with dietary reference value cut-points for vitamin C. Mean intakes of vitamin C from diet only (i.e., excluding supplements but including fortified foods for example breakfast cereals) were calculated for the whole population as well as for age and sex subgroups using the NDNS survey sampling weights provided. Calculations were made of the percentage of individuals meeting the RNI (estimated to meet the needs of 97.5% of the population), Lower Reference Nutrient Intake (LRNI; estimated to meet the needs of 2.5% of the population) and Estimated Average Requirement (EAR; estimated to meet the needs of 50% of the population).

### Food group categorisation

The 61 NDNS food categories (including beverages and supplements) were condensed into 33 summary food groups. No original food category was excluded due to uncertainty surrounding the vitamin C content within pre-determined food groups. An aggregate quantity of vitamin C intake for each category was calculated, from which mean daily intakes and percentage contribution of different food categories to vitamin C intakes were derived.

### Fruit juice categories

Hence, from the NDNS food file, the following sub-categories were included: Apple and blackcurrant fruit juice, not from concentrate, apple juice unsweetened canned, apple juice unsweetened cartons pasteurised, apple juice unsweetened UHT, apple/pear Juice concentrate unsweetened, blueberry, apple and grape fruit juice 100% juice, carrot juice cartons or bottles, frozen orange juice (OJ) concentrate, fruit juice fortified with multivitamins, grape juice carbonated (grape juice not canned), grapefruit juice unsweetened UHT, lemon juice 50% vit C loss, lemon juice only no peel or flesh, mango juice fresh, mixed fruit juice canned unsweetened 100% juice, mixed fruit juice pasteurised, OJ freshly squeezed, OJ sweetened bottle, OJ unsweetened ambient/UHT, OJ unsweetened pasteurised, Pineapple juice (PJ) sweetened bottled, PJ sweetened canned, PJ unsweetened canned, PJ unsweetened pasteurised, PJ unsweetened UHT, pomegranate juice purchased, prune juice bottled unsweetened, redcurrant juice fresh, smoothies red bottled, purchased fruit and juice blends (max 1 portion), tomato juice cartons or bottles, vegetable juice mixed, Vita fit multivitamin 11 fruit juice, wheatgrass juice.

### Smoothie categories

The food group “Smoothies” was composed of the following sub-category names: Smoothies yellow purchased, Smoothies yellow with coconut, Smoothie red purchased (and fortified), Smoothies green purchased, “Naked” mango machine smoothie, “Naked” blueberry machine smoothie, “Naked” strawberry, raspberry and cranberry juice smoothie.

### Dietary misreporting

Dietary misreporting was estimated using the Goldberg cut-off (11), i.e., the ratio of reported energy intake (EI) to Basal Metabolic Rate (BMR), where BMR was calculated using the Oxford Equation (12). Predicted physical activity levels (PAL), as set out by SACN (13) were utilised as follows: 1–<3 y 1.40 (median PAL); 2–<10 y 1.58 (median PAL); 10–18 y 1.75 (median PAL); 19 y + 1.63 (median PAL). A physical activity level of 1.63 for adults represents a low activity level and has been utilised in previous NDNS datasets to estimate average energy requirements for adults.

Additionally, the percentage difference in reported EI and Estimated Energy Requirements (EER) was determined by the following equation: EI reported (kcal/Day) – EER/EER ×100 (where BMR was calculated as above utilising Henry equations (2005) and EER for each subject was determined as BMR × PAL as per ratios set out above).

### Dietary modelling amounts of fruit juice on intakes of vitamin C

NDNS data files as described above were manipulated in replacement scenarios to model what effect varying the amount of FJ consumed, or adding new consumers, would have on intakes of vitamin C and free sugars. Dietary records (mostly 4 days and no less than 3 days) were altered, either by varying the amounts of FJ where these were reported as consumed, or adding FJ into the diets of a percentage of those who did not report consuming any FJ during the dietary reporting period.

A Stochastic modelling technique was used where a mathematical model was constructed using the survey data. This was used to estimate the potential change in vitamin C status due to varying intakes of FJ and assess the impact when an increased intake of fruit juice is balanced by a decreased intake of another calorically comparable food and vice versa. The effect of varying FJ was calculated in each diet, with EI replacement coming from either the individual’s other foods or fruit or sugar beverages as consumed by other individuals. Since the main energy source of the beverages is sugar, fruit juices and fruit beverages have similar energy content (14). Evidence also suggests that FJ increases the total energy intake, with the same effect as other beverages such as cola or milk (15). For the purpose of the modelling, a number of assumptions were made. In some scenarios, where there was a random element to the diet changes, we repeated the scenario 30 times selecting different consumers completely at random from the population of respondents and averaged the results. The standard error was then obtained from the variation in these 30 simulations and the variation among the individuals sampled by the survey. Modelling was done using the R statistical programming language version 4.4.2 (R Foundation for Statistical Computing, Vienna).

### Fruit juice replacement scenarios

The modelling process involved taking respondents reported intake records and modifying them. The daily FJ consumption of all individuals in the database who reported consuming FJ was varied in intervals of 10% from −100 to +100%. The actual consumption (for example 50 mLs FJ) was used and the modelled amount was 0–100 mLs. There was no assumption that additional FJ was in a single serving, however it was assumed that it was consumed sometime over the 4-day recording period and the time pattern of consumption will not affect the nutritional calculations.

The category of FJ, in this scenario and all others, was extended to include fruit smoothies, but to exclude consumption of very small amounts of FJ (<20 kcal) which in almost all cases, related to lemon juice. EI had to remain constant, therefore when FJ was reduced it was assumed that participants consumed a small amount of other foods they already consumed to remain isocaloric. Thus, replacements for this energy were made with either: (a) all the other foods in the survey that the same individual respondent reported consuming, with small increases or decreases in the amounts of each, (b) replacing FJ with fruit and (c) replacing FJ with sugar sweetened beverages (SSB). Note that options (b) and (c) were only suitable for scenarios where FJ consumption was reduced. When FJ was replaced with fruit or SSB, the specific items were selected completely at random from the intake records of all participants of these items. When replacing with fruit, a random sample of all fruits consumed across the population was used; no specific replacement of juice with the corresponding whole fruit was substituted. When replacing with SSB, this was replaced with a random sample of all types of SSB consumed across the population.

These approaches ensured that the distribution of fruit or food items selected matched those currently consumed. It was this random selection element which was simulated 30 times.

### Fruit juice non-consumers scenarios

For this scenario, a random sample was selected of between 10 and 50% of non-consumers of FJ, and we added FJ to their dietary records. The added daily FJ was selected from a randomly selected person of the same age group and sex, and the selection was repeatedly simulated 30 times. The amounts of all other foods reported by each sampled person were adjusted by a small amount so that the respondents total EI remained the same. The individuals for which FJ was added were selected at random, and so each scenario between 10 and 50% was replicated 30 times and the nutritional results were averaged.

## Results

Within the 3,558 NDNS participants, 1,424 (40%) reported consuming FJ during the 4-day survey period (<10 years *n* = 194: 11–18 years *n* = 152: 19–64 *n* = 816: >65 = 262) while 94 individuals (<3%) reported consuming smoothies (<10 years *n* = 29: 11–18 years *n* = 19: 19–64 *n* = 38: >65 = 6). Notably, despite the low number of consumers, smoothie consumption ranged from 13 g/day–795 g per day with an overall mean of 211 g/day (SD 133 g/day).

The top 5 contributors to overall vitamin C intakes are presented in Table 1 by age group. The leading contributor to vitamin C intake (% contribution) in the total population was FJ at 25% (40% consumers), followed by fruit (24.2%; 82% consumers), cooked vegetables (24%; 96% consumers), smoothies (21.6%; 2.7% consumers) and low-calorie soft drinks (12.5%; 44% consumers). Within each age group, the top vitamin C contributors were as follows: Children aged 1–3 y: Fruit (29.4%; 94% consumers); Children aged 4–10 y: FJ (29.5%; % consumers 47%); Adolescents aged 11–14 y: FJ (35.5%; 50% consumers); Adolescents aged 15–18 y: FJ (32.5%; 44% consumers); Adults aged 19–64y: Cooked vegetables (26.2%; 97% consumers); Adults aged 65-75y: Fruit (28.1%; 86% consumers); Older adults aged 75+: FJ (29.5%; 34% consumers).

**Table 1:** Mean percent contribution of smoothies, 100% fruit and/or juice and other key food groups to average vitamin C intakes (*n* 3,558).

Age	Smoothies			Fruit Juice			Cooked vegetables			Soft drinks low calorie			Fruit		
	*n*	% consumers	% contribution	*n*	% consumers	% contribution	*n*	% consumers	% contribution	*n*	% consumers	% contribution	*n*	% consumers	% contribution
ALL (*n* 3,558)	94	3	21.6	1,424	40	25.0	3,422	96	24.0	1,556	44	12.5	2,922	82	24.2
1–3 (*n* 109)	8	7	16.4	46	42	24.4	101	93	11.7	67	61	20.2	103	94	29.4
4–10 (*n* 314)	22	7	20.1	148	47	29.5	296	94	14.8	215	68	17.9	288	92	22.3
11–14 (*n* 167)	10	6	27.7	83	50	35.5	157	94	14.9	103	62	13.8	135	81	18.6
15–18 (*n* 155)	9	6	26.4	69	44	32.5	142	92	19.4	78	50	12.7	105	68	19.4
19–64 (*n* 2,156)	39	2	22.9	816	38	23.3	2,082	97	26.2	934	43	11.8	1,730	80	24.2
65–74 (*n* 399)	4	1	11.3	175	44	19.1	396	99	26.4	107	27	5.1	344	86	28.1
75+ (*n* 258)	2	1	12.8	87	34	29.5	249	97	26.6	54	21	5.6	217	84	24.0

%, percentage; cal, kilocalorie; % consumers determined by number of consumers/total population (3,558). % contribution determined by vitamin C intake of food group/total intake of vitamin C. Data presented as mean percentages. Food was categorised for each food group as follows: Low calorie Soft Drinks included all low calorie, no sugar, sugar free drinks including carbonated or still and inclusive of low-calorie energy drinks. Smoothies included all fortified and non-fortified pureed fruit drinks including yellow, green, red smoothies as well as Naked mango, blueberry and strawberry, raspberry and cranberry juice. Fruit juice included fruit juices not from concentrate, unsweetened canned, unsweetened pasteurised, UHT and unsweetened concentrate as well as fruit juice fortified with multivitamins, carbonated grape juice, vegetable juice including tomato and wheatgrass juice. Also inclusive of lemon juice (no peel or flesh) and red smoothies with fruit and juice blends. Fruit included raw, baked, stewed, dried fruits and sauces, and canned fruit in juice and water. Cooked vegetables included fresh, cooked, tinned, pureed beans and legumes, raw, canned (added salt/no salt), fresh, pickled, frozen (boiled), pureed, roasted (in oil, Olive oil, vegetable oil, PUFA, butter, Ghee, Lard) vegetables.

Table 2 shows the study sample characteristics with mean (SD) age, sex split, and average daily intake of energy (Kcals), macronutrient intake (% energy), % free sugar and vitamin C of all included NDNS participants. More than half (51%) of participants were female. Overall daily vitamin C intake was 81.7 mg/day with the lowest vitamin C intake observed for children aged 1–3 y (64.2 mg/day) and the highest for older adults aged 65–74 y at 85.8 mg/day. Percent free sugar ranged from 9.7–12.3% across all age groups with the highest intakes reported for both younger and older adolescents (12.3% for both 11–14 y and 15–18 y).

**Table 2:** Demographic characteristics of NDNS participants from years 9–11 (*n* 3,558).

Variable	Total (*n* 3,558)	1–3 (years) (*n* 109)	4–10 (years) (*n* 314)	11–14 (years) (*n* 167)	15–18 (years) (*n* 155)	19–64 (years) (*n* 2,156)	65–74 (years) (*n* 399)	75+ (years) (*n* 258)
Age	40.73 ± 22.74	2.16 ± 0.79	7.05 ± 2.07	12.39 ± 1.14	16.45 ± 1.02	41.45 ± 12.80	69.58 ± 2.86	80.20 ± 3.51
Sex, *n* (%)								
Male	1,754 (49.3)	56 (51.3)	161 (51.2)	90 (54.0)	75 (48.4)	1,074 (49.8)	188 (47.1)	111 (43.0)
Female	1,804 (50.7)	53 (48.7)	153 (48.8)	77 (46.0)	80 (51.6)	1,083 (50.2)	211 (52.9)	147 (57.0)
Total Energy (kcal)	1,720 ± 539	1,056 ± 238	1,442 ± 339	1,622 ± 413	1,698 ± 500	1,828 ± 567	1,673 ± 449	1,589 ± 436
%TEI from carbohydrate	46.1%	49.1%	51.0%	50.4%	49.3%	45.0%	43.9%	46.4%
%TEI from Protein	16.2%	15.7%	14.8%	15.5%	15.9%	17.0%	17.0%	16.2%
%TEI from Fat	34.2%	35.3%	34.2%	34.2%	34.1%	34.1%	34.3%	34.6%
Protein (g)	70.16 ± 23.97	41.01 ± 10.07	52.94 ± 13.82	61.91 ± 16.67	67.20 ± 24.23	75.96 ± 25.08	69.31 ± 17.65	63.36 ± 16.95
Fat (g)	65.92 ± 25.21	41.48 ± 11.53	55.13 ± 16.29	62.19 ± 20.40	64.94 ± 23.62	69.83 ± 26.50	64.56 ± 23.53	61.79 ± 22.66
Carbohydrate (g)	209.71 ± 71.02	138.40 ± 35.75	195.82 ± 47.61	217.19 ± 56.51	221.83 ± 65.11	218.71 ± 77.36	193.65 ± 57.02	194.17 ± 53.23
Total sugars (g)	84.01 ± 38.91	62.26 ± 22.69	80.62 ± 26.30	81.37 ± 34.18	83.61 ± 36.92	86.31 ± 42.26	80.61 ± 34.95	85.26 ± 34.22
Free Sugars (g)	47.90 ± 32.28	27.94 ± 15.87	47.31 ± 21.68	53.57 ± 28.59	56.25 ± 32.31	49.71 ± 35.43	40.84 ± 25.90	44.15 ± 25.88
% Free sugars	10.2%	9.7%	12.1%	12.3%	12.3%	9.9%	9.0%	10.2%
Vitamin C (mg)	81.67 ± 53.54	64.25 ± 34.29	74.88 ± 39.35	77.93 ± 50.65	74.14 ± 49.67	84.69 ± 57.03	85.82 ± 53.69	72.55 ± 44.65

mg, milligrams; g, grams; kcal, kilocalories; %TEI, mean percentage total energy intake; %Free sugars, mean percentage of free sugars from total energy. All data presented as Mean ± standard deviation or number and percentage. Cases weighted utilising wti_9–11.

Table 3 presents vitamin C intakes by age. Median (IQR) intakes of vitamin C met or exceeded the RNI across all ages. Notably, the minimum values across all age ranges indicated lower than adequate intakes in some individuals. Additionally, the placing of the 5th and 10th percentiles suggested that a small section of the population may have inadequate intakes. The 75th percentile onwards and the maximum intakes demonstrate that some individuals are well above the RNI which is suggestive of a varied intake pattern or high consumption of rich vitamin C food sources since these data excluded supplements. Table 4 displays the proportion of individuals across the age groups and by sex who met the RNI, who fell below the EAR and subsequently those who also fell below the LRNI, and therefore at risk of deficiency for vitamin C. In the total population, 80.4% of men and 79.7% of women met the RNI for vitamin C; 7.7% (both males and females) fell below the EAR while the proportion of those falling below the LRNI was minimal (2% or less across all age groups).

**Table 3:** Total vitamin C intake from diet only for the age groups 1–3, 3–10, 11–14, 15–18, 19–64, 65–74. 75 + derived from NDNS rolling data years 9–11.

Age group (years)	*N*	Mean ±SD	Median (IQR) (mg)	Min	Max	Lower percentile			Upper percentile			RNI (mg)
						5th	10th	25th	75th	80th	95th	
1–3	109	64.25 ± 34.29	57.06 (223.47)	9.73	233.19	21.50	25.85	38.29	82.75	89.33	123.74	30
4–10	314	74.88 ± 39.35	68.62 (285.03)	5.41	290.44	23.90	31.17	45.67	96.06	105.14	152.72	30
11–14	167	77.93 ± 50.65	69.10 (395.53)	7.30	402.83	18.01	25.53	43.94	100.35	107.59	172.42	35
15–18	155	74.14 ± 49.67	61.73 (312.56)	2.06	314.62	20.24	25.04	38.05	97.12	110.44	167.55	40
19–64	2,156	84.69 ± 57.03	73.31 (588.65)	2.55	591.20	19.81	28.22	44.59	112.01	122.43	181.83	40
65–74	399	85.82 ± 53.69	72.46 (323.40)	9.37	332.76	21.68	30.14	47.41	111.52	126.41	187.27	40
75+	258	72.55 ± 44.65	60.45 (206.99)	6.77	213.76	19.47	24.79	40.02	94.73	109.10	165.71	40

mg, milligram; IQR, Interquartile range; RNI, Reference Nutrient Intake. RNI concentrations vary by age range; SD, standard deviation.

**Table 4:** Proportion of the United Kingdom population meeting dietary reference value thresholds for vitamin C.

Variables	Sex	TotalTotal	Years						
			1–3	4–10	11–14	15–18	19–64	65–74	75+
Meeting RNI threshold	Male	1,410 (80.4%)	49 (88.1%)	149 (92.9%)	73 (81.0%)	56 (74.3%)	846 (78.8%)	155 (82.4%)	82 (73.9%)
	Female	1,437 (79.7%)	46 (86.0%)	134 (87.9%)	64 (83.0%)	58 (73.0%)	857 (79.1%)	166 (78.8%)	112 (76.2%)
Below EAR	Male	135 (7.7%)	2 (4.0%)	3 (2.1%)	9 (9.7%)	6 (7.8%)	94 (8.8%)	10 (5.4%)	11 (9.5%)
	Female	139 (7.7%)	2 (3.8%)	6 (4.2%)	4 (5.5%)	9 (11.5%)	86 (7.9%)	15 (7.2%)	15 (10.4%)
Below LRNI	Male	10 (0.6%)	0 (0%)	0 (0%)	0 (0%)	0 (0%)	7 (0.7%)	1 (0.6%)	1 (0.7%)
	Female	18 (1.0%)	0 (0%)	1 (0.5%)	0 (0%)	2 (2.1%)	13 (1.2%)	0 (0%)	2 (1.5%)

RNI, Reference Nutrient Intake; LRNI, lower reference nutrient intake; EAR, estimated average intake, taken from: Department of Health, Dietary Reference Values for Food Energy and Nutrients for the United Kingdom, HMSO, 1991. Dietary Reference Value cut-offs vary by age ranges as follows: 1–10 years, LRNI = 8 mg/day, EAR = 20 mg/day, RNI = 30 mg/day; 11–14 years, LRNI = 9 mg/day, EAR = 22 mg/day, RNI = 35 mg/day; 15–75 + years, LRNI = 10 mg/day, EAR = 25 mg/day, RNI = 40 mg/day.

Data excludes (*n*=409) who did not meet the RNI threshold but whose intake remained above the EAR therefore considered to be marginally at risk of inadequate intake.

Table 5 presents the estimate of misreporting within the total population according to age group as indicated by the Goldberg cut-off and % EER methods. Overall average EI: BMR ratio was estimated at 1.14 and 1.17 for males and females, respectively. A total of 38.8% males and 37.5% of females had an EI: BMR ratio below 1.1 indicative of implausible reporting. The % difference between reported EI and EER (%EER) was calculated as −28.68% and −26.55% for males and females, respectively. The extent of underreporting among both male and female adolescents aged 15–18y was highest compared with all other age groups. Specifically, 54% of males and 45.8% of females aged 15–18y fell below the EI: BMR cut off. Correspondingly, %EER for males of this age was −40.17% while for females was −37.6%.

**Table 5:** Misreporting within total population per physical activity level cut-offs.

Age group (years)	EI: BMR	Males			EI: BMR	Females		
		Plausible	Below cut-off value 1.1	% misreporting		Plausible	Below cut-off value 1.1	% misreporting
Total	1.14	Plausible	680 (38.8%)	−28.68	1.17	Plausible	677 (37.5%)	−26.55
1.5–3	1.58	Plausible	3 (5.7%)	12.49	1.30	Plausible	9 (17.4%)	−7.08
4–10	1.82	Plausible	10 (6.5%)	15.39	1.31	Plausible	24 (15.6%)	−16.47
11–14	1.14	Plausible	42 (46.5%)	−34.95	1.18	Plausible	29 (37.2%)	−32.44
15–18	1.05	Implausible	41 (54.0%)	−40.17	1.09	Plausible	37 (45.8%)	−37.60
19–64	1.16	Plausible	461 (43.0%)	−28.85	1.16	Plausible	462 (42.7%)	−29.00
65–74	1.16	Plausible	75 (40.0%)	−28.83	1.17	Plausible	80 (37.7%)	−28.11
75 +	1.11	Plausible	47 (42.6%)	−31.81	1.20	Plausible	36 (24.7%)	−26.33

All data presented as median. EI: BMR = ratio of reported energy intake (EI) to estimated Basal metabolic rate (BMR), used to assess dietary reporting plausibility. A cut-off value of <1.1 (based on the Goldberg method) was used to indicate under-reporting. Estimated energy requirements were calculated as BMR x physical activity level (PAL), using the age appropriate BMR equations and assumed PAL values. Physical activity level set for each age category: 0–3 y, 1.40; 3–10 y, 1.58; 10–18 y, 1.75; 18+, 1.63. Percentage misreporting (% misreporting) reflects the median percentage difference between reported energy intake (EI) and estimated energy requirements (EER) calculated as: (Ei-EER)/EER × 100. Negative values indicate under-reporting of energy intake.

**Figure 1:**
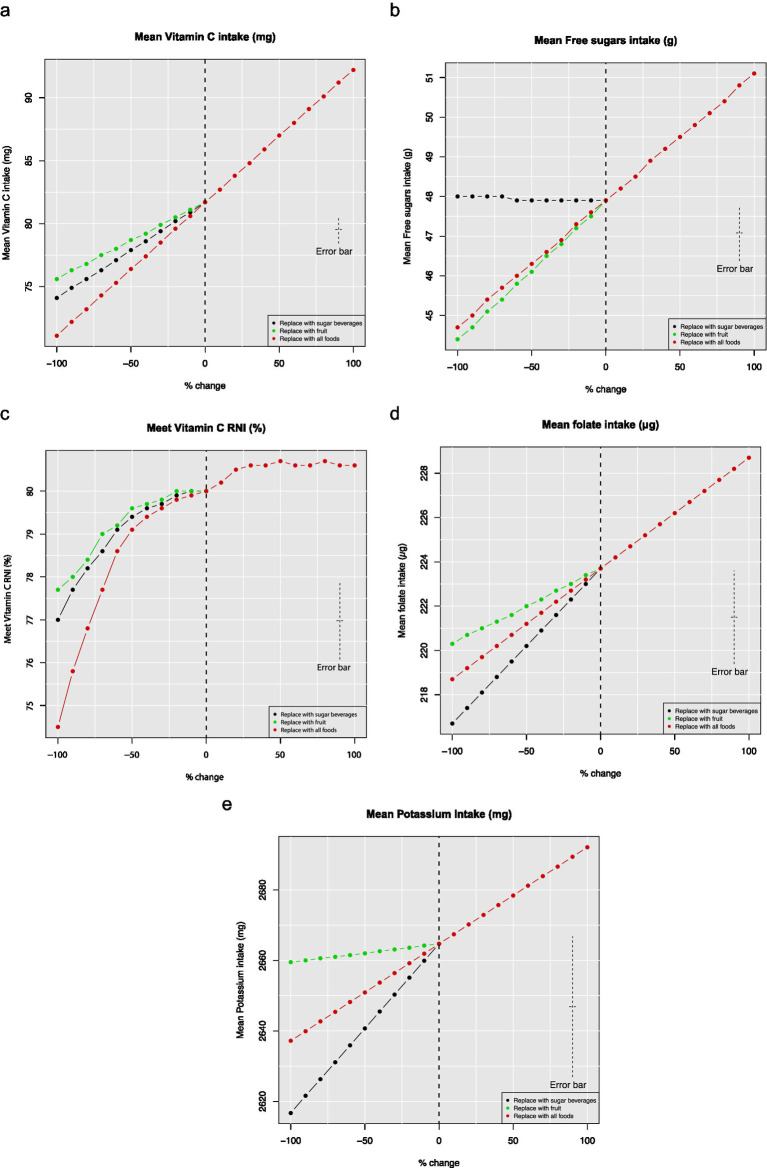
The impact of modelled reductions or increases in Fruit Juice on **(a)** Mean vitamin C intake (mg), **(b)** Mean free sugar intake (g), **(c)** Meeting Vitamin C RNI, **(d)** Mean folate intake (µg) and **(e)** Mean potassium intake (mg).

Mean vitamin C intake (mg/day) based on replacement scenarios with sugar beverages, fruit or all foods is shown in Figure 1a. If current FJ consumers reduced their intake it would have a modest effect on vitamin C intakes, from a current average of about 82 mg/day to 71–76 mg/day. This occurred in the majority of consumers (*p* < 0.001). However, the extent of the reduction would depend on what the FJ was replaced with, to maintain the same amount of energy in the diet and avoid body weight change. Replacement with more of the other foods the person already consumed would produce the biggest reduction, whereas if it was replaced with additional fruit or with SSB (many of which have some vitamin C added) the reduction would be less. Figure 1b shows that a reduction of FJ intake would lead to a small (<10%) reduction in free sugar intakes, from about 48 g/day to 44 g/day (<0.001) in almost everyone, if FJ is replaced with fruit or with other foods, except in a very few individuals whose other foods had very high free sugars and so this increased when they replaced FJ with these foods. No reduction in free sugars would occur if FJ was replaced with SSB. Increasing FJ in current consumers would lead to a similarly small increase in free sugar intake.

If current consumers increased their intake of FJ, then mean vitamin C intakes would increase. If intakes of FJ doubled (from an overall mean of approx. 100 g/day to 200/day,), mean vitamin C would increase to about 92 mg/day. The effect on the proportion meeting RNI is much less, an increase of less than 1% (Figure 1c). This is presumably because most FJ consumers already meet RNI with their current intakes.

Increasing the proportion of FJ consumers in the overall sample showed that this would theoretically increase the mean intake of vitamin C. If 20% of non-consumers started consuming FJ, with similar mean intakes to current consumers, then mean intakes of vitamin C would increase to 87 mg/day. For 50% of non-consumers, this would become 94 mg/d. Here, it was assumed that adding FJ to the diet would lead to a matched reduction in an average of the other foods to keep EI constant.

The impact of modelled reductions or increases in FJ on folate intakes is much more modest, with mean intakes of 224 mg/d reducing to 216–217 mg/d with removal of FJ then increasing to 229 mg/day with the addition of FJ (Figure 1d). Replacement with fruit or other foods in general leads to a smaller reduction than with replacement by SSB. A similar conclusion of very small effects is observed in potassium intakes (Figure 1e).

## Discussion

This secondary analysis reported vitamin C intakes and food sub-category contributors for a representative sample of the United Kingdom population. Additionally, using dietary modelling, the theoretical impact on vitamin C intakes of varying the amounts of FJ in consumers or by adding FJ into the diets of non-consumers was examined. Across all age groups, vitamin C intakes from foods and beverages (excluding supplements) typically exceed the current United Kingdom RNI (30-40 mg/day), with adults aged 19–64y achieving the highest mean intakes, at over 84 mg/day and the lowest in adults >75 years at 73 mg/day. Importantly, a small proportion of the United Kingdom population fall below the EAR for vitamin C: predominantly, male adults aged 75 + (10.4%), and teenage girls aged 15–18 y (11.5%), while the proportion of those falling below the LRNI was <2% across all age groups and within the expected tolerance of <2.5%. The present study therefore has found that United Kingdom males and females of all ages are at a low risk of vitamin C deficiency.

The current global Recommended Daily Allowance (RDA’s) and Nutrient Reference Values (NRV) for vitamin C vary widely between countries, primarily due to some countries using a minimum amount to prevent deficiency as in the United Kingdom, and other countries using an appropriate amount to maintain vitamin C status and optimise health (3). In the United States the RDA are 90 mg/day and 75 mg/day for men and women, respectively, (16) whilst the United Kingdom current recommendation (established in 1991) is 40 mg/day for adults. For comparative purposes, under food labelling legislation within the EU and United Kingdom, the Nutrient Reference Value for vitamin C is 80 mg [regulation EU, (17) no 1169/2011]. Notably, a recent commentary reports strong evidence that optimal vitamin C intakes vary among individuals and that United Kingdom vitamin C recommendations should be re-evaluated (18).

The leading contributors to vitamin C intakes in this United Kingdom population, were FJ, fruit, cooked vegetables and smoothies. In the case of smoothies, less than 3% of the population in 2016–19 were found to be consumers, however excessive intakes of up to 795 g per day were observed within the consumers which may explain this finding. Despite the relatively modest amounts consumed (just over 100 mLs/day on average), FJ contributes 25% to average vitamin C intakes across all ages within this United Kingdom population, marginally exceeding contributions from cooked vegetables (24%) and fruit (24.2%). These contributions were based on the 40% of the United Kingdom population who reported FJ consumption, considerably lower than for other vitamin C sources including fruit and cooked vegetables (82% & 96% consumers respectively). Furthermore, consumption patterns are age dependent. For example, teenagers aged 11–14 y were most likely to consume FJ (50%), compared to 38% of adults aged (19–64 y), in line with that reported in Ireland ((39%); (9)). Among adults in the current study the main source of vitamin C was cooked vegetables (26% contribution). Notably, over 34% of older adults aged 75+, reported FJ consumption and, as such, FJ was the leading contributor to vitamin C intake in this age group, contributing 29.5%. It is also noteworthy that, while generally sufficient at just over 64 mg/day, vitamin C intakes for younger children (aged 1–3 y) were the lowest compared to all other age groups, with fruit identified as the main source of vitamin C in this age group. Nevertheless, with respect to vitamin C contributions, the current study shows that FJ makes important contributions to dietary intakes of vitamin C across all age groups (24–35.5% of intake) in the United Kingdom. This is consistent with data reported in consumption surveys across 9 WHO European Regions (9) which indicated that FJ contributed up to one-quarter of intakes of vitamin C ranging from 4–26% across countries and ages (9). However, fruit juice contributed 54% of total vitamin C intakes in Brazil and 37% in the US (19). Notably, on average one small glass of 100% commercial and fresh juices as sold in the United Kingdom provides 67.3 mg of vitamin C, which comfortably exceeds the RNI and hence would be considered to contain nutritionally significant levels of vitamin C (20).

Dietary modelling was used in the current study to evaluate the effects on vitamin C intakes if more of the population consumed FJ, or if current consumers increased the amount of FJ consumed whilst remaining isocaloric. In the scenario where intakes of FJ doubled (from an overall mean of approx. 100 mLs/day to 200 mLs/day), mean vitamin C would increase to approximately 92 mg/day. If consumers vary their intake of one of the few foods containing significant amounts of vitamin C it is inevitable that vitamin C intake will vary. Similarly, in the scenario where additional FJ consumers were added, an increase in the mean intakes of vitamin C was observed, with the pattern of changes similar across age and sex groups. Specifically (and assuming that additional intake of FJ leads to a matched reduction in all other foods), where 20% of non-consumers started consuming FJ with intakes similar in distribution to current consumers, mean vitamin C intakes in the United Kingdom population would increase to 87 mg/day. In the case of 50% of non-consumers becoming consumers, mean vitamin C would rise to 94 mg/d. As expected, a much greater effect on the proportions of people meeting dietary reference value thresholds of adding consumers than of increasing the amount of FJ consumed in current consumers was observed. This is logical given the high proportion (80.4% males; 79.7% females) of the total United Kingdom population already meeting the RNI for vitamin C. However concerningly, 7.7% of the population fell below the EAR and <2% fell below the LRNI which identifies those not likely to meet their needs for vitamin C and at risk of deficiency (4), with evidence suggesting that there is substantial variation in the proportions of people with low vitamin C intakes from different social groups (18). These findings highlight a potential need for dietary education to support at risk sub groups on increasing rich sources of vitamin C, within the context of a healthy diet.

Modelling used in this study assumed that any change in fruit juice consumption is accompanied by compensatory changes in consumption of other foods so that energy intake remains the same and the diet is isocaloric. Authors acknowledge that such complete compensation may be unlikely, and in reality, it would only be partial. If so, the biggest effect of FJ consumption changes would be weight gain or loss. For example, 100 mL of fruit juice (estimated 50 calories) consumed 4 times per week is estimated to provide approximately an additional 200 kcal per week, which would increase energy intake. Incomplete compensation in other foods is unlikely to change the results much, since the proportion of calories in FJ is relatively small, and so a few percent more or less in total intake would not appreciably change this.

However, in this no-compensation situation the effect on vitamin C and other nutrients would be very similar to what is presented for the scenario where all other foods are adjusted in compensation. This is because the amount of adjustment is small relative to total energy, and replacement is mostly with the many other components of the diet (cereals, protein sources etc) which have little impact on vitamin C. The other two scenarios (replacement with sugar beverages or with fruit) are included to examine the situation where fruit juice is changed by those making considered choices about their diets. If fruit is regarded as “better” than fruit juice for example, someone currently consuming fruit juice for health reasons might make that change. Alternatively, if FJ is regarded as “better” than sugar sweetened beverages, then some might opt for the latter as they are more widely available and there is more variety. It is for these reasons that we present these 3 scenarios: fruit juice changes are accompanied by changes in other foods (which will be similar to no other changes) or in fruit or in sugar beverages. It is clear that regardless of whether FJ is added or substituted for another food/drink, there appears to be minimal impact on energy balance. However in public health terms, it would be advisable to recommend that FJ replaces sugar-sweetened beverages such as carbonated drinks, nectars, juice drinks or cordials, rather than replacing whole fruit.

Currently 150 mLs/day of FJ/smoothies counts towards one serving of the recommended 5-a-day target within United Kingdom food based dietary guidelines (21) and this is the maximum which should be consumed to avoid excess free sugar consumption. Dietary guidelines for FJ are not uniform between countries, and the definition of FJ can vary outside of the EU/United Kingdom (22). In the United Kingdom and EU, it is not permitted to add sugar to FJ, therefore the sugar content cannot be modified and is representative of the natural sugar content of the intact fruit (23). In contrast, nectars and other fruit drinks are permitted to contain added sugars and/or sweeteners yet are sometimes categorised alongside FJ in dietary surveys, making it difficult to separate the nutritional impact of FJ alone (9). Confusion also arises with the definition of free sugars because this category includes both added sugars and those naturally present in FJ, honey and syrups (SACN, 2015), meaning that FJ may be grouped for policy reasons with soft drinks with added sugars. However, 100% FJ is typically low in energy and contains key micronutrients including vitamin C and folate, potassium as well as bioactive substances (e.g., polyphenols, carotenoids and pectin) and natural fruit sugars. Indeed, consumption of 100% FJ is reported to have beneficial effects on some cardiometabolic health outcomes, with evidence from a systematic review and meta-analysis of prospective cohort studies suggesting that the beneficial effect on CVD risk may be due to a positive impact on blood pressure, arterial compliance and endothelial function (24). This was confirmed in a systematic review and meta-analysis of prospective studies and randomised controlled trials of FJ consumption which also found no consistent evidence that FJ consumption is associated with an increased risk of overweight or excess body weight gain (25). Similarily a scoping review for Nordic Nutrition recommendations suggest that moderate intakes of 100% fruit juice are not associated with obesity or chronic disease risk and may have a protective effect on cardiovascular disease (33) and similarilry 100% fruit juice did not show any beneficial effects on the risk of developing T2DMd (26).

However, it is known that %100 fruit juice is lower in dietary fibre relative to whole fruit, and it is frequently cited that this may impact on satiety which may adversely impact on energy intake and weight management (27). Additionally due to its high in naturally occurring sugars, some evidence suggests that it has a negative health effect.

Due to its categorisation as a source of free sugars, the role of FJ in a healthy diet remains controversial, with studies reporting that FJ intake is associated with higher intakes of total and free sugars (28). A recent review (9) found that across the lifecycle in Europe, FJ contributed 2–14% of free sugars but made negligible contributions to total energy intakes (<3%) and importantly made small contributions to intakes of folate and potassium (<7%) and contributed up to a quarter (4–26%) of vitamin C intakes. In the current study, diet modelling showed that when typical fruit juice intake was reduced and replaced with fruit or other foods (foods already consumed within the NDNS population), only very small reductions (by <5 g/day) in free sugar intakes occurred. Likewise increasing fruit juice in current consumers resulted in only a small increase in mean free sugar intake (about 1.5 g/day with a 50% increase in FJ intake or 3 g/day with a 100% increase in FJ). The reduction in free sugars was only very slightly more when FJ was replaced with fruit than with other foods (modelling used foods already being consumed by the population). These findings provide evidence that the impact on overall free sugar intakes of moderate fruit juice consumption in line with current recommendations is minimal.

Currently in the United Kingdom, average intakes of fruits and vegetables are low, and too few adults and young people meet the 5-a-day recommendations (5). In the current study, daily consumption of one portion of 100% FJ (ranging from approx 70 g/day to 158 g/day) was shown to be one possible strategy to improve vitamin C adequacy and could be useful in helping to achieve 5-a-day recommendations, minimally impacting free sugar intakes and providing key micronutrients alongside whole fruit and vegetables. Importantly, there was more benefit to vitamin C intakes if a greater proportion of people drank the recommended serving of FJ (150 mLs) rather than current consumers drinking more FJ, thus supporting the current United Kingdom dietary guidelines on FJ. Notably, our modelling exercise found that if current FJ consumers reduced their intake of FJ, it would have a modest effect by reducing vitamin C intakes, from a current average of about 82 mg/day to 71–76 mg/day. The extent of the reduction would depend on what FJ was replaced with, and also the need to maintain the same amount of energy in the diet to avoid weight change. Replacement with more of the other foods (not fruit & vegetables) the person already consumed would produce the biggest reduction, whereas if it was replaced with additional whole fruit or beverages fortified with vitamin C the reduction would be smaller. However, the substitution of FJ with drinks of lower to no nutritional quality, for example SSB, would negatively impact on the overall quality of the diet and would not be recommended as part of a healthy diet (PHE, 2016).

Limitations of the current findings include that the analysis was conducted on United Kingdom NDNS data collected from 2016–2019, and before publication of the most up to date NDNS data (2019–2023). As such the dietary patterns presented may not represent the most recent United Kingdom food intake patterns, albeit the data are representative of intake patterns that were not impacted by the COVID pandemic which resulted in changes to typical dietary habits and food choices in the United Kingdom (29). It is worth noting that the NDNS rolling programme is based on a random sample of average (mean/median) daily 3–4 day intakes which likely vary more than long term individual averages. As such, the between individual variability may be overestimated. This means that those individuals with higher intakes than the upper limit or below the RNI may be overestimated. Approximately 30% underreporting of energy intake was noted in the current study, which is in line with previous population-based research (30, 31). In this study the level of underreporting was higher among adolescents, indicating that actual vitamin C intakes may be higher than reported with consequences for the previously reported DRVs, reducing the proportion of individuals below the RNI. These findings highlight the importance of identifying misreporters in national food intake data, however removal of participants who were likely to be under-reporters of energy intake would have greatly impacted the sample size available for the current study. Notably, dietary modelling was entirely projected based on systematic or complete replacement of 100% FJ with quantities scaled, therefore the effects of substitution are theoretical. Finally, it is important to consider the global variation in RDAs for vitamin C which will impact the interpretation of the findings in a wider context. Specifically, the current United Kingdom RDA of 30-40 mg/day, established in 1991, and predominantly based on the prevention of scurvy, has come under recent scrutiny for being too low based on newer evidence relating to broader clinical outcomes (18).

In conclusion, the results of the present study indicate that FJ contributes 25% to overall vitamin C intake in consumers across a representative sample of the United Kingdom population, and current consumers of FJ are almost all meeting DRV’s for vitamin C. However just 40% of this population are identified as consuming FJ. Notably adolescents aged 11–14 years were the highest consumers (50%) of FJ which provided 35.5% of overall vitamin C in this age group and in light of the high levels of misreporting observed in this age group actual intakes are potentially higher. While increasing the amount of FJ consumed may increase typical population vitamin C intakes, this would not change the numbers of people meeting the DRV, and importantly amounts in excess of 150 mLs per day are not recommended in order to mitigate free sugar consumption. Encouraging more people, especially low consumers of whole fruit, to include a maximum of one daily 150 mL serving of FJ in their diet, is an accessible strategy to achieve the national “5-a-day” recommendation for fresh fruit and vegetables, as currently recommended in national dietary guidelines.

At these low and moderate intake levels (<150 mLs/day), 100% juice can be incorporated into a healthy, balanced diet without the risks associated with excess consumption of free sugars and energy, however regular high intakes are likely to impact negatively on overall diet quality.

## Data Availability

The present study utilised food and nutrient intake data from years 9 to 11 (2016–2019; n 3,558) which were obtained from the United Kingdom Data Archive (NatCen, University of Essex, Colchester, Essex, United Kingdom). Further inquiries can be directed to the corresponding author/s.
